# HIV-1 drug resistance among HIV/HCV co-infected patients with treatment failure in Yunnan, southwestern China: a cross-sectional study

**DOI:** 10.3389/fmicb.2026.1715352

**Published:** 2026-03-10

**Authors:** Huan Li, Xiaobo Ma, Ya Li, Chenglu He, Fang Zou, Xilin Kang, Min Zhong

**Affiliations:** 1 Department of Clinical Laboratory, The First Affiliated Hospital of Kunming Medical University, Kunming, Yunnan, China; 2 Yunnan Province Clinical Research Center for Laboratory Medicine, Kunming, Yunnan, China; 3 Yunnan Key Laboratory of Laboratory Medicine, Kunming, Yunnan, China; 4 Kunming Medical University, Kunming, Yunnan, China; 5 Department of Clinical Laboratory, Yan’an Affiliated Hospital of Kunming Medical University, Kunming, Yunnan, China

**Keywords:** drug resistance, genotype, highly active antiretroviral treatment, HIV-human immunodeficiency virus, HIV/HCV co-infection

## Abstract

**Background:**

Yunnan Province remains a region with a high prevalence of human immunodeficiency virus (HIV) in China. Due to shared transmission routes, HIV/Hepatitis C virus (HCV) co-infection is common. This study aimed to analyze the prevalence and mutation patterns of HIV-1 drug resistance among HIV/HCV co-infected patients specifically following the virological failure of first-line highly active antiretroviral therapy (HAART) in Yunnan, and to compare these characteristics with HIV-1 mono-infected patients.

**Methods:**

A cross-sectional study was conducted among 104 HIV/HCV co-infected and 120 HIV-1 mono-infected patients who experienced virological failure (HIV-RNA\geq 1,000 copies/mL) after at least 6 months of ART. Genotypic drug resistance was tested using an in-house method and analyzed via the Stanford HIV drug resistance database. Multivariable logistic regression and stratified analysis were performed to adjust for confounders.

**Results:**

Among patients with treatment failure, the drug resistance rate in the HIV/HCV co-infection group (37.5%) was significantly lower than in the HIV-1 mono-infection group (55.0%, *P* = 0.009). Multivariable logistic regression showed that HIV/HCV co-infection was associated with a lower trend of resistance, although it did not reach formal statistical significance after adjusting for gender, treatment duration, and CD4 + Count (aOR = 0.49, *P* = 0.084). However, stratified analysis revealed that co-infection was significantly associated with lower resistance in patients with a treatment duration of 6–12 months (OR = 0.24, *P* = 0.001) and those with CD4 + Count ≤ 350 cells/μL (OR = 0.38, *P* = 0.001). The frequency of the NRTI-related mutation T69D/N/S was significantly lower in the co-infected group (*P* = 0.029).

**Conclusion:**

Among patients experiencing virological failure, HIV/HCV co-infection is associated with distinct genotypic resistance profiles, particularly in the early stages of treatment failure and among immunodeficient individuals. These findings suggest that co-infection status may influence the pathway to HIV drug resistance. Clinicians should prioritize prompt genotype resistance testing for co-infected patients failing ART to optimize second-line regimen adjustments.

## 1 Introduction

Acquired immunodeficiency syndrome (AIDS) is an infectious disease created by human immunodeficiency virus (HIV) characterized by serious damage to immune system activity. It has seriously threatened human survival and had a great impact on global population health. Hepatitis C virus (HCV) belongs to the flaviviridae family, and its transmission route is the same as that of HIV. Therefore, HIV/HCV co-infection cases are common among HIV-infected patients. Recent data show that more than 35 million people worldwide are infected with HIV, while more than 150 million people are infected with HCV (Gobran et al., 2021). It is reported that approximately 25–50% of HIV-1-infected individuals globally are co-infected with HCV (Bouare et al., 2012; D’Aleo et al., 2017), while in China, the co-infection rate ranges from 18.2 to 48.67% (Wu et al., 2017; Zhang et al., 2014), and the co-infection rate in Kunming (Yunnan) is 19.4% (Li et al., 2018). The incidence of HIV/HCV co-infection varies greatly among different populations. Study has shown that the co-infection rate caused by the blood route can reach more than 50% (Prussing et al., 2015), which is significantly higher than that of patients infected through sexual contact. In particular, the co-infection rate of HIV/HCV in AIDS patients with intravenous drug addiction or blood infection is 100 and 73.3%, respectively (Zheng et al., 2017).

The widespread epidemic of HIV resistant strains caused by antiretroviral therapy is a global public health challenge. The antiretroviral treatment of AIDS in developed countries has started earlier, and there are more studies on HIV drug resistance during treatment. During the active replication of the HIV, the pro-virus clearly records the various states of the viral genome. During highly active antiretroviral therapy in first use, antiretroviral drugs mutate the target genes on HIV, resulting in the drug-resistant strains becoming the dominant strains, and the spread of drug-resistant strains is on the rise. Multiple drug resistance often occurs in clinical diagnosis and treatment. Moreover, drug resistance between different kinds of drugs often crosses, which increases the difficulty of inhibiting virus replication. Retrovirus drug resistance is an important public health problem, because it limits treatment options, leads to treatment failure and HIV rapid spread, and affects future treatment options for untreated patients (Saravanan et al., 2012).

Due to the geographical location bordering many countries, the number of AIDS patients in Yunnan has always been in the forefront of the country, which has seriously affected the health of the local people and hindered the economic development of Yunnan. Since the highly active antiretroviral therapy in first use was launched in 1996, a great progress has been made in the treatment of AIDS. However, due to the same transmission route, co-infection cases of HIV and HCV are common. For a long period of time in the future, the HIV/HCV co-infected epidemic strains in China will mutate and evolve under the pressure of increasing and more complex drugs. The purpose of this study is to detect HIV-1 genotype drug resistance in HIV/HCV co-infected patients who have received highly active antiretroviral therapy in first use treatment but have failed virologically, and to explore the occurrence and characteristics of secondary drug resistance in HIV/HCV co-infected patients, so as to provide a theoretical basis for the diagnosis, treatment and prevention of HIV/HCV co-infected patients.

## 2 Materials and methods

### 2.1 Study design

A cross-sectional study was conducted in Yunnan Province, China. 224 Patients at the First Affiliated Hospital of Kunming Medical University who had treatment failure after receiving first-line anti-retroviral therapy for more than 6 months from 2019 to 2022 were enrolled. Although this was a single-center study, the patients were recruited from a major referral hospital in Kunming, which serves a diverse population across Yunnan Province. Our cohort included individuals with diverse transmission routes, primarily intravenous drug use and sexual contact, reflecting the key epidemiological patterns of this high-prevalence region.

### 2.2 Ethical approvals

This study was approved by Medical Ethics Committee of First Affiliated Hospital of Kunming Medical University, Kunming, China (approval ID: 2018-L-24). All patients signed written informed consent to participate in this study.

### 2.3 Patients and plasma

This study specifically enrolled HIV-1 infected patients who had experienced virological failure, defined as having an HIV-1 viral load ≥ 1,000 copies/mL after receiving first-line ART for at least 6 months. By focusing on this treatment-experienced population with inadequate viral suppression, we aimed to compare the resistance profiles of those with and without HCV co-infection.

#### 2.3.1 Inclusion criteria

Voluntary signing of informed consent, HIV antibody was screened and confirmed positive, HIV-RNA viral load ≥ 1,000 copies / mL. The patients were treated with first-line anti-retroviral therapy, Treatment time ≥ 6 months, HCV antibody and HCV-RNA were positive (HIV/HCV co-infected patients).

#### 2.3.2 Exclusion criteria

Patients with opportunistic infection occurred within 14 days, Patients with AIDS related malignant tumors and blood diseases, Patients with digestive tract diseases such as acute and chronic pancreatitis, Patients with severe mental or neurological diseases, Female patients during pregnancy and lactation, Patients who are not from Yunnan.

A total of 285 HIV-infected patients receiving ART at the First Affiliated Hospital of Kunming Medical University were retrospectively screened between 2019 and 2022. To ensure the study specifically addressed drug resistance in the context of treatment failure, 26 patients were excluded for having an HIV-1 viral load < 1,000 copies/mL. An additional 18 patients were excluded due to missing plasma samples or insufficient volume for successful “in-house” genotyping. Based on the defined exclusion criteria, 17 further cases were removed, including 7 pregnant or lactating women, 5 patients with severe mental or neurological diseases, 3 non-Yunnan residents, and 2 patients with opportunistic infections within 14 days. Ultimately, 224 patients meeting the definition of virological failure were enrolled, comprising 104 HIV/HCV co-infected and 120 HIV mono-infected individuals.

### 2.4 Sample collection and processing

Ten milliliter venous blood was collected in negative pressure blood collection vessel containing EDTA anticoagulant. The detection of T cell subsets is completed within 4 h, after that, the plasma is separated and stored at -80°C. Avoid repeated freezing and thawing of samples.

### 2.5 Detection of T lymphocyte subsets

T lymphocyte subsets was detected by BD FACS Count reagent kit (BD, United States) and BD FACS Canto II flow cytometer (BD, United States) following the manufacturer’s instructions.

### 2.6 Detection of HIV-1 viral load in plasma

HIV-1 viral load in plasma was assessed using a HIV-RNA quantitative detection kit (Roche, Switzerland) and following the manufacturer’s instructions. The Gene amplification instrument is COBAS AmpliPrep COBAS TaqMan 48 (Roche, Switzerland).

### 2.7 Detection of HIV-1 genotyping drug resistance

Plasma RNA was extracted by QIAamp Viral RNA Mini Kit (Qiagen, Germany) and GeneRotex automatic nucleic acid extractor (Tianlong, China) according to the manufacturer’s instructions. The reverse transcription reaction were performed on the extracted RNA using TaKaRa One Step RNA PCR Kit (AMV). The primers used are shown in Supplementary Table 1. The reaction system and procedure are carried out with reference to the literature (Li et al., 2011). The Real-Time-PCR product was subjected to 1% agarose gel electrophoresis. The recovered fragments were entrusted to Beijing Nuosai Gene Co., Ltd. for sequencing. The sequencing primers were shown in Supplementary Table 1. A total of 5 HIV pol gene sequences were tested for each sample, including 2 reverse and 3 forward sequences. The sequencing results were spliced by ContigExpress. After splicing, the sequences were sorted and proofread by BioEdit. Finally, the results were uploaded to Stanford HIV drug resistance database^1^ for drug resistance mutation analysis. The sequencing results of PR and RT gene were analyzed to obtain the resistance and resistance sites of protease inhibitor and reverse transcriptase inhibitor. A score corresponding to each drug is also obtained according to the drug resistance mutations, and finally the drug resistance is judged according to the cumulative total score. If the result was not “sensitive,” it was judged to be drug resistant.

### 2.8 Statistical analysis

Statistical analysis was performed using SPSS software version 22.0 (IBM Corp. Released 2013. IBM SPSS Statistics for Windows, Version 22.0. Armonk, NY: IBM Corp.). *P* < 0.05 were regarded as statistically significant. The qualitative data was expressed as number (n) and percentage (%). χ^2^-test was used for pairwise analyses of qualitative data. GraphPad Prism software version 8 was used for data visualization.

## 3 Results

### 3.1 Clinical information of patients

104 HIV/HCV co-infected patients and 120 HIV-1 mono-infected patients with HIV-1 viral load ≥ 1,000 copies/mL who received highly active antiretroviral therapy in first use for over 6 months in Yunnan Province were enrolled. Both groups of patients were diagnosed from 2019 to 2022.The average ages of HIV/HCV co-infected patients and HIV-1 mono-infected patients were 38 ± 6 years and 40 ± 13 years, the median viral load was 13,846 copies/mL and 14,450 copies/mL, the median CD4 + T lymphocytes were 218 and 235/mL, respectively. The drugs used by the patients were recommended by the Manual of China National Free Antiretroviral Treatment Program (Cai et al., 2016). The clinical information of patients are shown in Table 1. As the results showed that intravenous drug addiction is the main mode of transmission in HIV/HCV co-infected patients.

**TABLE 1:** Clinical information of 104 HIV/HCV co-infected patients and 120 HIV-1 patients (χ^2^-test).

Clinical information		HIV/HCV co-infected patients (*N* = 104)	HIV-1 mono-infected patients (*N* = 120)	*P*
Gender	Male	89 (85.6%)	67 (55.8%)	**< 0.001**
	Female	15 (14.4%)	53 (44.2%)	
Nationality	Han	90 (86.5%)	109 (90.8%)	0.373
	Minority	14 (13.5%)	11 (9.2%)	
Route of transmission	Sexual intercourses transmission	12 (11.5%)	88 (73.3%)	**<0.001**
	Intravenous drug addiction	90 (86.6%)	21 (17.5%)	
	Unknown	2 (1.9%)	11 (9.2%)	
HIV genotype	CRF07_BC	28 (26.9%)	18 (15%)	**<0.001**
	CRF08_BC	71 (68.3%)	74 (61.7%)	
	CRF01_AE	2 (1.9%)	18 (15%)	
	B	3 (2.9%)	10 (8.3%)	
Treatment period (month)	6–12	40 (38.5%)	60 (50%)	0.228
	13–24	34 (32.6%)	27 (22.5%)	
	25–36	11 (10.6%)	15 (12.5%)	
	>36	19 (18.3%)	18 (15%)	
Medication	D4T/AZT+3TC+NVP	56 (53.8%)	78 (65%)	0.089
	D4T/AZT/TDF+3TC+EFV	48 (46.2%)	42 (35%)	

Bold values represent statistically significant.

### 3.2 Drug resistance mutations of HIV/HCV co-infected patients

A total of 59 HIV/HCV co-infected patients detected drug resistance-related mutations. Among the 59 cases, the most common mutation related to protease inhibitors (PIs) was V82F, accounting for 1.0% (1/104), followed by the mutation L10I/V/IL [4.8% (5/104)], A71T/AT/V [4.8% (5/104)], and V11I [1.0% (1/104)].Among the mutations associated with nucleoside reverse transcriptase inhibitors (NRTIs), the most frequent mutations were T69D/N/S [15.4% (16/104)] and M184V/I [13.5% (14/104)].Among the mutations associated with NNRTIs, the most frequent mutation sites were K103N/KN/KR/Q/NS [22.1% (23/104)] and V179E/DV/DEV/T [13.5% (14/104)], and the frequency of G190A /GRS/AG and Y181C were all 5.8% (6/104), and the frequency of H221Y/HY was 4.8% (5/104). The details of other drug resistance mutations are shown in Table 2.

**TABLE 2:** Drug resistance mutations in 59 HIV/HCV co-infected patients.

Case number	Drug resistance mutation of PIs	Drug resistance mutation of NRTIs	Drug resistance mutation of NNRTIs
HIV/HCV-1	L10I	M41ILM, D67N, K70R, M184V, T215F, K219Q	K103KN, Y181C
HIV/HCV-3			A98G, K103N, Y181C, G190A, H221Y, K238T
HIV/HCV-6	L10V	M184V	K103N, G190AG
HIV/HCV-8	A71AT		K103N
HIV/HCV-12		T69S	V179DEV
HIV/HCV-13	V82F, V11IV	M184V	
HIV/HCV-15			K103N
HIV/HCV-16		T69S	K103KN
HIV/HCV-17		M184V	
HIV/HCV-18			V106M, Y188C
HIV/HCV-21		T69S, K70KR, M184V	H221HY
HIV/HCV-26			V106A, V179D, P225H
HIV/HCV-29		T69S	V179D
HIV/HCV-31			V179D
HIV/HCV-32	L10I		
HIV/HCV-35			E138A
HIV/HCV-44			K103N
HIV/HCV-45			V179T
HIV/HCV-48	A71V		V179DEV
HIV/HCV-49			V90I, K103N, Y181C, H221HY
HIV/HCV-50		T69S	
HIV/HCV-51		M184I	
HIV/HCV-52		M184V	V90IV, K103N, V179E
HIV/HCV-53			Y188L
HIV/HCV-54		M41L, V75I, M184V, L210W, T215Y	V90IV, V108I, Y181C, H221Y
HIV/HCV-55	A71AT		
HIV/HCV-60	L10V		
HIV/HCV-61	A71T		
HIV/HCV-62	A71V		
HIV/HCV-64		A62V, T69N	K103KR
HIV/HCV-65			V179D
HIV/HCV-67			E138A
HIV/HCV-68			K103N
HIV/HCV-69		A62V, T69N	K103KN, V179DV, G190GRS
HIV/HCV-70			
HIV/HCV-74		T69D	K103N, E138A
HIV/HCV-76		T69S	
HIV/HCV-78		T69S	
HIV/HCV-79		A62V, T69D, M184V	Y181C
HIV/HCV-80			K103N, V108IV
HIV/HCV-82			K103N, V179E
HIV/HCV-84	L10IL	M184V	K103NS, G190AG
HIV/HCV-85		K219KR	K103KN
HIV/HCV-86			G190A
HIV/HCV-88		T69S	V179D
HIV/HCV-91			K103N, P225HP
HIV/HCV-92			V179D
HIV/HCV-93			L100ILM, K103N, P225HP
HIV/HCV-94		T69S	
HIV/HCV-96		A62V, T69N	K103KN
HIV/HCV-97			
HIV/HCV-98		M184V	K103N
HIV/HCV-99		T69S, M184V	K103Q, V106M, F227FL
HIV/HCV-101		M184V, T215Y	V90IV, H221HY
HIV/HCV-102			V179D, Y181C, G190A
HIV/HCV-103		M184V	V179D
HIV/HCV-106			
HIV/HCV-107			K103N, P225H, K238N
HIV/HCV-108		T69S	

### 3.3 Antiretroviral drug resistance in HIV/HCV co-infected patients

**FIGURE 1:**
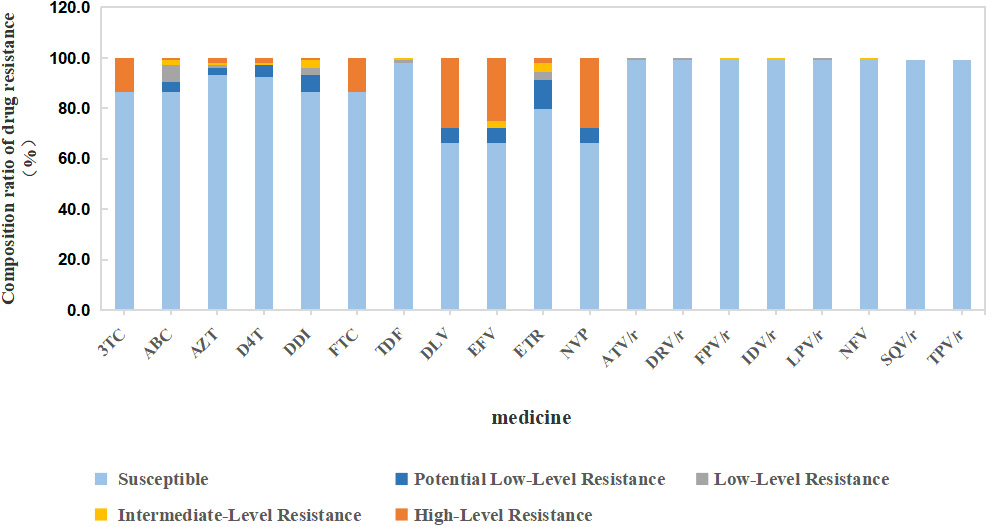
Anti-retroviral drug resistance in HIV/HCV co-infected patients (*n* = 104). 3TC, lamivudine; ABC, abacavir; AZT, zidovudine; D4T, stavudine; DDI, di-deoxyinosine; FTC, emtricitabine; TDF, tenofovir; DLV, delavirdine; EFV, efavirenz; ETR, etravirine; NVP, nevirapine; ATV/r, atazanavir/ritonavir; DRV/r, darunavir/ritonavir; FPV/r, fosamprenavir/ritonavir; IDV/r, indinavir; LPV/r, lopinavir/ritonavir; NFV, nelfinavir/ritonavir; SQV/r, saquinavir/ritonavir; TPV/r, tipranavir/ritonavir.

Among the 104 HIV/HCV co-infected patients, 39 patients had different degrees of drug resistance. Among the used NNRTIs, the proportions of high resistance to nevirapine (NVP) and efavirenz (EFV) were 27.9 and 25.0%, respectively. Among the nucleoside drugs, the proportions of high resistance to lamivudine (3TC), zidovudine (AZT), and stavudine (D4T) were 13.5, 1.9, and 1.5%, respectively (Figure 1). Among the 39 patients with drug resistance, 19 patients were resistant to non-nucleoside reverse transcriptase inhibitors (NNRTIs), 3 patients were resistant to NRTIs, 16 patients were resistant to both NRTIs and NNRTIs, and only 1 patient had low-level resistance to protease inhibitors.

### 3.4 Comparison of HIV-1 genotype drug resistance between HIV/HCV co-infected patients and HIV-1 mono-infected patients

The drug resistance rates of 104 patients with HIV/HCV co-infection and HIV-1 mono-infection were 37.5% (39/104) and 55.0% (66/120), respectively. The drug resistance rate of HIV/HCV co-infection group was lower than that of HIV-1 mono-infection group (χ^2^ = 6.852, *P* = 0.009). The results showed that the incidence of drug resistance in female patients and Han patients in HIV/HCV co-infection group was lower than that in HIV-1 mono-infection group (χ^2^ = 5.972, 4.594, *P* = 0.015, 0.032). The patient’s CD4+T cells > 350 means that there is only mild immunodeficiency, and a value of 350 or less means that the patient has moderate immunodeficiency, at which time the patient has developed more opportunistic infections and tumor lesions. Patients with HIV RNA viral load > 100,000 copies/mL after treatment indicated that the treatment effect was poor and the virus was not effectively controlled. Typically, after effective therapy, patients should have a significant decrease in HIV RNA viral load, usually below the lower limit of detection or at least below 100,000 copies/mL. If the viral load persists above this level, it may be because the virus has become resistant to the drugs in the treatment. Within the variables of treatment period within 1 year, the treatment regimen containing NVP, CD4+ T lymphocytes ≤ 350 cells/μL, HIV-1 viral load ≤ 100,000 copies/mL, there was significant difference in the incidence of drug resistance between HIV/HCV co-infection group and HIV-1 mono-infection group (χ^2^ = 10.774, 4.060, 10.376, 4.063, *P* = 0.001, 0.044, 0.001, 0.044). There was no significant difference in the incidence of drug resistance among different infection routes (all *P* > 0.05), as shown in Table 3.

**TABLE 3:** Comparison of drug resistance between HIV/HCV co-infected patients and HIV-1 patients (χ^2^ test).

Variable		HIV / HCV co-infected patients (*N* = 104)	HIV-1 mono-infected patients (*N* = 120)	*P*
		Number of drug-resistant cases(%)	Number of drug-resistant cases(%)	
Gender	Male	35(39.3)	33 (49.3)	0.550
	Female	4(26.7)	33 (62.3)	**0.015**
Nationality	Han ethnic	35(38.9)	59 (54.1)	**0.032**
	Ethnic minorities	4(28.6)	7 (63.6)	0.080
Route of transmission	Intravenous drug addiction	34(37.8)	8 (38.1)	0.978
	Sexual intercourses transmission	4 (33.3)	54 (61.4)	0.065
	Unknown	1 (50.0)	4(36.4)	0.715
Treatment	D4T/AZT/TDF+3TC+EFV	18 (37.5)	23 (54.8)	0.101
	D4T/AZT+3TC+NVP	21 (37.5)	43 (55.1)	**0.044**
Treatment period (month)	6–12	10 (25.0)	35 (58.3)	**0.001**
	13–24	16 (47.1)	18 (66.7)	0.126
	25–36	5 (45.5)	10 (66.7)	0.279
	>36	8 (42.1)	3 (37.5)	0.824
Number of CD4^+^T lymphocytes (cells/μL)	≤350	31 (35.6)	56 (59.6)	**0.001**
	>350	8 (47.1)	10 (38.5)	0.576
HIV-RNA(copies/mL)	≤ 100,000	31 (34.4)	58 (54.7)	**0.044**
	>100,000	8 (57.1)	8 (57.1)	1.000

Bold values represent statistically significant.

There was significant difference in the incidence of drug resistance mutations between HIV/HCV co-infected patients and HIV-1 mono-infected patients, which were 56.7 and 70.8%, respectively (χ^2^ = 4.826, *P* = 0.028. Drug resistance related mutations were detected in 59 cases of HIV/HCV co-infection group and 85 cases of HIV-1 mono-infection group. Two cases of multi-drug resistance mutations were detected in HIV/HCV co-infection group. There were 16 cases in the HIV/HCV co-infection group with dual resistance mutation sites related to NRTIs and NNRTIs, while 32 cases were detected in the HIV-1 infection group. Therefore, the frequency of dual drug resistance mutation in HIV/HCV co-infection group was significantly lower than that in HIV-1 mono-infection group (χ^2^ = 4.212, *P* = 0.04). The drug resistance mutation K219Q/KR related to NRTIs, the mutations K238T/N, L100ILM, and F227FL related to NNRTIs, and the mutations V82F and V11IV related to PIs were only detected in the HIV/HCV co-infection group, while the mutations K65R, Y115F, K101E/H, M230L, I50IV, M46IM, Q58E were only detected in HIV-1 infection group. Compared with HIV-1 infection group, the frequency of drug resistance mutation sites associated with NRTIs in HIV/HCV co-infection group was significantly different (χ^2^ = 7.845, *P* = 0.005), especially the frequency of mutation T69D/N/S in HIV/HCV co-infection group was significantly lower than that in HIV-1 mono-infection group (χ^2^ = 4.785, *P* = 0.029), while there was no significant difference in drug resistance mutations related to PIs and NNRTIs between two groups (all *P* > 0.05) (see Table 4).

**TABLE 4:** Comparison of drug resistance mutations between HIV/HCV co-infected patients and HIV-1 patients (χ^2^ test).

Mutation site	HIV/HCV co-infected Patients (*N* = 104) %(number of cases)	HIV-1 mono-infected patients (*N* = 120) %(number of cases)	*P*
PIs related:	10.5 (11)	10.8 (13)	0.951
V82F	1.0 (1)	0	
A71T/AT/V	4.8 (5)	5.0 (6)	
L10I/V/IL	4.8 (5)	1.7 (2)	
V11IV	1.0 (1)	0	
I50IV	0	0.8 (1)	
M46IM	0	0.8 (1)	
Q58E	0	0	
NRTIs related:	26.5 (28)	45.0 (54)	0.005
M184I/V	13.5 (14)	20.8 (25)	
A62V	3.8 (4)	5.8 (7)	
M41L/LM	1.9 (2)	1.7 (2)	
L210W	1.0 (1)	0.8 (1)	
V75I	1.0 (1)	3.3 (4)	
D67N	1.0 (1)	4.2 (5)	
T69D/N/S	15.4 (16)	27.5 (33)	**0.029**
K70R/KR	1.9 (2)	0.8 (1)	
T215Y/F	2.9 (3)	3.3 (4)	
K219Q/KR	1.9 (2)	0	
K65R	0	2.5 (3)	
Y115F	0	0.8 (1)	
NNRTIs related:	41.3 (43)	49.2 (59)	0.241
A98G	1.0 (1)	0.8 (1)	
G190A/GRS/AG	5.8 (6)	11.7 (14)	
K103N/KN/KR/Q/NS	22.1 (23)	21.7 (26)	
Y181C	5.8 (6)	6.7 (8)	
K238T/N	1.9 (2)	0	
H221Y/HY	4.8 (5)	0.8 (1)	
E138A	2.9 (3)	5.0 (6)	
V179E/DV/DEV/T	13.5 (14)	13.3 (16)	
V90I/IV	3.8 (4)	0.8 (1)	
L100ILM	1.0 (1)	0	
P225H/HP	3.8 (4)	4.2 (5)	
V108I/IV	1.9 (2)	0.8 (1)	
V106M/A	2.9 (3)	4.2 (5)	
Y188C/L	1.9 (2)	5.8 (7)	
F227FL	1.0 (1)	0	
K101E/H	0	5.0 (6)	
M230L	0	2.5 (3)	

Bold value represents statistically significant.

4.3 In the HIV/HCV co-infection group, 19 cases were resistant to NRTIs (18.3%) Among them, 3 cases only showed drug resistance to NRTIs (2.9%), 16 cases were resistant to both NRTIs and NNRTIs (15.4%). There are 35 cases were resistant to NNRTIs (33.7%). Among them, 19 patients were only resistant to NNRTIs (18.3%), and only 1 patient was resistant to PIs. In HIV-1 infection group, 36 cases were resistant to NRTIs (30%), 57 cases were resistant to NNRTIs (47.5%), 29 cases were resistant to both NRTIs and NNRTIs (24.2%), and 5 cases were resistant to PIs. There was significant difference in the efficacy of first-line drugs NRTIs and NNRTIs after the failure of anti-viral treatment between HIV/HCV co-infection group and HIV-1 mono-infection group (χ^2^ = 4.139, 4.413, *P* = 0.042, 0.036) (Table 5).

**TABLE 5:** Comparison of drug resistance between HIV/HCV co-infected patients and HIV-1 patients (χ^2^ test).

	HIV/HCV co-infected patients (*N* = 104)					HIV-1 mono-infected patients (*N* = 120)					*P*
Resistance medicine	Sensitive	Potential low-level	Low-level	Intermediate-level	High-level	Sensitive	Potential low-level	Low-level	Intermediate-level	High-level	
PIs:	103	1 (0.1%)				115	5 (4.2%)				0.138
ATV/r	103		1	1		119	1	1		1	
DRV/r	103		1			110	1				
FPV/r	103		1			118		1			
IDV/r	103			1		119	1				
LPV/r	103			1		118	1				
NFV	103					116	2	2			
SQV/r	103					120	2				
TPV/r	103					118					
NRTIs:	85	19 (18.3%)				84	36 (30%)				**0.042**
3TC	90	4	7	2	14	92	7	14	3	25	
ABC	90				1	92			5	2	
AZT	97	3	1	1	2	110	6	1	2	2	
D4T	96	5		1	2	103	8		6	2	
DDI	90	7	3	3	1	90	14	8	6	2	
FTC	90		1	1	14	92	1	1	3	25	
TDF	102					114			3	1	
NNRTIs:	69	35 (33.7%)				63	57 (47.5%)				**0.036**
DLV	69	6	3	3	29	66	6	1	4	43	
EFV	69	6			26	68	4	5	12	31	
ETR	83	12		4	2	79	17	10	8	6	
NVP	69	6			29	67	4	5	1	43	

Bold values represent statistically significant.

To further investigate the relationship between HIV/HCV co-infection and drug resistance while controlling for potential confounders, a multivariable logistic regression analysis was performed. The model included gender, nationality, transmission route, ART regimen, treatment duration, CD4+ count, and viral load as covariates. Although HCV co-infection showed a trend toward a lower likelihood of drug resistance, the association did not reach formal statistical significance in the overall adjusted model (aOR = 0.49, 95% CI: 0.22–1.10, *P* = 0.084) (Table 6). However, stratified analysis revealed that the impact of co-infection status varied significantly across different clinical subgroups (Table 7). Specifically, HIV/HCV co-infected patients had significantly lower odds of drug resistance compared to mono-infected patients among those with a treatment duration of 6–12 months (OR = 0.24, 95% CI: 0.10–0.57, *P* = 0.001) and those with moderate to severe immunodeficiency (CD4+ count ≤ 350 cells/μL, OR = 0.38, 95% CI: 0.21–0.69, *P* = 0.001). A marginal difference was also observed among patients infected via sexual transmission (*P* = 0.065). These results suggest that while the overall independent effect of co-infection is nuanced, its association with lower resistance rates is most pronounced during the early stages of treatment failure and in patients with lower CD4+ T lymphocyte counts.

**TABLE 6:** Multivariable logistic regression analysis of factors associated with HIV-1 drug resistance.

Variable	Adjusted odds ratio (aOR)	95% Confidence interval (CI)	*P*
HCV co-infection (vs. HIV mono-infection)	0.49	(0.22, 1.10)	0.084
Gender (female vs. male)	1.07	(0.55, 2.10)	0.837
Nationality (minority vs. han)	1.12	(0.43, 2.91)	0.814
Transmission route (IVDU vs. sexual)	0.63	(0.28, 1.40)	0.254
ART regimen (NVP-based vs. EFV-based)	1.02	(0.57, 1.83)	0.95
Treatment duration (13–24 months vs. 6–12 months)	1.86	(0.93, 3.72)	0.081
Baseline CD4+ count (≤350 vs. > 350 cells/μL)	0.96	(0.46, 2.02)	0.911
HIV viral load (>100,000 vs. ≤ 100,000 copies/mL)	1.94	(0.80, 4.73)	0.143
Variable	Adjusted odds ratio (aOR)	95% Confidence interval (CI)	*P*
HCV co-infection (vs. HIV mono-infection)	0.49	(0.22, 1.10)	0.084
Gender (female vs. male)	1.07	(0.55, 2.10)	0.837
Nationality (minority vs. han)	1.12	(0.43, 2.91)	0.814
Transmission route (IVDU vs. sexual)	0.63	(0.28, 1.40)	0.254
ART regimen (NVP-based vs. EFV-based)	1.02	(0.57, 1.83)	0.95
Treatment duration (13–24 months vs. 6–12 months)	1.86	(0.93, 3.72)	0.081
Baseline CD4+ count (≤350 vs. > 350 cells/μL)	0.96	(0.46, 2.02)	0.911
HIV viral load (>100,000 vs. ≤ 100,000 copies/mL)	1.94	(0.80, 4.73)	0.143

**TABLE 7:** Stratified analysis of factors associated with HIV-1 drug resistance.

Variable stratification	Co-infected group resistance rate	Mono-infected group resistance rate	Odds ratio (OR)	95% confidence interval (CI)	Statistical significance
**Transmission route**					
- IV drug use (IVDU)	37.80%	38.10%	0.99	(0.37, 2.62)	Non-significant
- Sexual transmission	33.30%	61.40%	0.31	(0.09, 1.13)	Marginal (*P* = 0.065)
**Treatment duration**					
- 6–12 months	25.00%	58.30%	0.24	(0.10, 0.57)	Significant (*P* = 0.001)
- 13–24 months	47.10%	66.70%	0.44	(0.16, 1.26)	Non-significant
- > 36 months	42.10%	37.50%	1.21	(0.22, 6.61)	Non-significant
**Baseline CD4 + count**					
- ≤ 350 cells/μL	35.60%	59.60%	0.38	(0.21, 0.69)	Significant (*P* = 0.001)
- > 350 cells/μL	47.10%	38.50%	1.42	(0.41, 4.90)	Non-significant

## 4 Discussion

Drug resistance remains a primary challenge in AIDS treatment. It arises from mutations in the viral targets of antiretroviral drugs, which alter drug binding sites and reduce therapeutic efficacy (Li, 2010). The development of resistance is a complex process influenced by viral genetic diversity, replication dynamics, and the fitness cost of specific mutations (Li, 2010). HIV/HCV co-infection is common due to shared transmission routes, yet the virological and clinical interactions between these viruses, particularly regarding the development of HIV drug resistance following treatment failure, are not fully understood (Zhao and Fu, 2012).

Existing research on HIV/HCV co-infection has largely focused on viral genotypes (Avanzi et al., 2017; Liu et al., 2009; Rallón et al., 2012), treatment outcomes (Castells et al., 2017; Tang et al., 2015; Younossi et al., 2015), and molecular epidemiology (Nazziwa et al., 2020; Pimentel et al., 2020). In contrast, studies specifically comparing genotype resistance profiles between co-infected and mono-infected individuals after virological failure are less common (Wensing et al., 2017; Ross et al., 2018; Sui et al., 2014). Our analysis addresses this gap. The observed drug resistance rate of 37.5% in our co-infected Yunnan cohort aligns with the rate (40.6%) reported by Wang among co-infected people who use drugs in Honghe and Dehong, Yunnan (Wang et al., 2018), and is consistent with our prior regional studies of treatment failure (Li et al., 2013). Notably, the distribution of resistance in our cohort differed, showing a higher proportion of patients with dual NRTI/NNRTI resistance or NNRTI-only resistance compared to the earlier report by Wang et al. (2018).

A key finding of this study is the significantly lower overall genotypic drug resistance rate observed in HIV/HCV co-infected patients compared to their HIV mono-infected counterparts. In our study, multivariable logistic regression analysis was employed to control for potential confounders such as gender, age, and treatment regimen. Although the adjusted odds ratio (aOR = 0.49) suggested that HCV co-infection might be a protective factor against HIV drug resistance, the association did not reach formal statistical significance (*P* = 0.084) (Table 6). This lack of significance may be partially attributed to the limited sample size, which reduced the statistical power to detect independent effects in the presence of multiple covariates. However, the consistent trend (aOR < 1) across models warrants further investigation in larger, prospective cohorts. Placing this result in a broader context reveals important contrasts. Some studies, such as those by Rocheleau et al. (2017) and Zhong et al. (2014), found no statistically significant difference in resistance rates between these groups, while Deng Haohui reported no significant difference in natural drug resistance (Deng et al., 2018). These discrepancies may be explained by variations in study design and population characteristics. Factors such as the proportion of people who inject drugs (which was high in our Yunnan cohort), the distribution of circulating HIV-1 subtypes (e.g., the predominance of CRF07_BC and CRF08_BC in this region), differences in first-line antiretroviral therapy protocols, and levels of medication adherence likely contribute to the observed differences in resistance epidemiology across studies. This underscores the necessity for region-specific resistance surveillance.

The results of this study carry direct implications for clinical practice in Yunnan and similar settings. First, they underscore the critical importance of prompt genotype resistance testing upon virological failure in all patients, with heightened awareness of the high prevalence of NNRTI resistance among co-infected individuals. Second, clinicians should be cognizant of the potentially different resistance pathways in co-infected patients when selecting second-line regimens, as the distinct mutation patterns (e.g., lower frequency of T69D/N/S) could influence the choice of alternative drugs. Finally, these findings strongly support advocating for integrated HIV/HCV testing, treatment, and management programs in Yunnan to address both infections comprehensively, which may ultimately contribute to improving treatment outcomes and containing the spread of drug-resistant viruses.

The finding from our stratified analysis (Table 7) was that the impact of HCV co-infection on HIV drug resistance is highly context-dependent. We observed a significant reduction in resistance rates among co-infected patients during the early stage of treatment failure (6–12 months, OR = 0.24) and in those with lower CD4+ counts ( ≤ 350 cells/μL, OR = 0.38). This suggests that the potential interference between HCV and HIV, possibly through viral competition for resources or modulation of host immune responses, may be most influential when the HIV viral population is under initial drug selection pressure or when the immune system is significantly compromised. As treatment duration extends beyond 24 months, this “interference” effect appears to diminish, possibly because long-term treatment failure leads to the inevitable emergence of resistant quasispecies regardless of co-infection status.

The observed differences in drug resistance profiles suggest the possibility of viral interference in HIV/HCV co-infection. This concept is supported by studies of other viral co-infections. For instance, research on GB Virus C (GBV-C), which shares structural and functional similarities with HCV, has demonstrated that viral proteins (e.g., E2 and NS5A) can inhibit HIV entry and replication through interactions with host cell receptors (e.g., CCR5, CXCR4) (Bagasra et al., 2012; Herrera et al., 2009; Herrera et al., 2011; Jassey et al., 2019; Koedel et al., 2011; Mohr et al., 2010; Xiang et al., 2012). Given the structural homology between GBV-C and HCV proteins, it is plausible that HCV co-infection might similarly modulate the host cell environment or HIV replication dynamics, potentially affecting the emergence of drug-resistant variants (Farnaz, 2011; Mohr et al., 2010). However, this remains a hypothesis. Our clinical and genotypic data do not provide direct mechanistic evidence. The putative role of HCV proteins (such as E2 or NS5A) in influencing HIV replication fitness and drug resistance evolution warrants specific investigation in future *in vitro* and molecular studies. There were differences between HIV/HCV co-infection group and HIV-1 mono-infection group in the variables of treatment time within 1 year, use of NVP-containing medicine, CD4+ T lymphocytes ≤ 350 cells/μL, and HIV viral load ≤ 100,000 copies/mL. The differences in these variables occurred during antiretroviral treatment, so it speculated that the similar effects produced by E2 and NS5A proteins of HCV during the treatment may also inhibit the mutation of HIV under drug selection pressure.

The incidence of drug resistance sites in 104 patients with HIV/HCV co-infection and HIV-1 mono-infection was 56.7 and 70.8%, respectively. The incidence of drug resistance sites in the two groups was significantly different (χ^2^ = 4.826, *P* = 0.028). The prevalence of NRTIs-related drug resistance mutations in HIV/HCV co-infection group was significantly higher than that in HIV-1 mono-infection group (χ^2^ = 7.845, *P* = 0.005). In particular, the frequency of T69D/N/S mutation in HIV/HCV co-infection group was significantly lower than that in HIV-1 mono-infection group (χ^2^ = 4.785, *P* = 0.029), while there was no significant difference in drug resistance mutation sites associated with PIs and NNRTIs (both *P* > 0.05).

At present, the national free antiretroviral treatment program is mainly a combination of a NNRTIs and a NRTIs. The results showed that the incidence of drug resistance to NRTIs and NNRTIs in the HIV/HCV co-infection group was 18.3% (19/104) and 33.7% (35/104), respectively, which was similar to the data reported by Wang Yu in the treatment group of intravenous drug users (Wang et al., 2018). The rate of resistance to both NRTIs and NNRTIs was 15.4% (16/104), and only 1 case was resistance to PIs. HIV/HCV co-infected patients have a higher drug resistance rate in first-line drugs due to the long-term use of reverse transcriptase inhibitors (including NRTIs and NNRTIs). The study showed that 16 out of 35 patients with NNRTIs resistance in the HIV/HCV co-infection group have dual resistance to NRTIs and NNRTIs, and more than 70% of them have high-level resistance to EFV and NVP. If these patients continue to use NRTIs and NNRTIs, they will eventually lead to ineffective treatment. Therefore, patients who fail to use the first-line antiviral treatment should carry out drug resistance monitoring as soon as possible, and then replace to the second-line drugs according to the drug resistance results, so as to improve the effect of antiretroviral treatment. And it was found that there were significant difference in the efficacy of first-line drugs between HIV/HCV co-infected group and HIV mono-infected group. Therefore, it is speculated that after HIV-infected patients are co-infected with HCV, the ability of drugs to enter HIV-infected cells is weakened, the genetic barrier to drug resistance is increased, and the replication adaptability of HIV is reduced (Hosseinipour et al., 2009).

Among HIV/HCV co-infected patients who failed antiretroviral treatment, the incidence of NNRTIs resistance was relatively high, and there were many drug resistance mutations, suggesting that NNRTIs resistance related mutation is a prominent problem in the antiretroviral treatment in Yunnan. Therefore, to strengthen the management of patients on antiretroviral therapy, we should start from enhancing the medication compliance and minimizing the occurrence of drug resistance, which will become the key to the success of antiretroviral therapy in Yunnan. In addition, if co-infection with other viruses may improve the effect of antiretroviral treatment, which will open up a new way for the research on HIV drug resistance. When choosing the antiretroviral treatment plan, drug resistance testing of patients should be carried out in time, and reasonable and effective first-line and second-line drugs should be used according to the drug resistance results to improve the survival rate of HIV-infected patients. In addition, drug-resistant strains produced by patients who failed antiretroviral treatment will increase the rate of HIV transmission and the risk of primary drug-resistance. Therefore, monitoring the occurrence of drug resistance in HIV-infected patients after antiretroviral treatment will not only improve the efficacy of the treatment, but also delay the spread of drug-resistant strains.

This study is limited by its retrospective design and the lack of systematically collected detailed clinical data, including WHO disease staging, specific opportunistic infections, documented adverse drug reactions, and standardized adherence metrics. These unmeasured factors may influence treatment outcomes and resistance development, and their absence limits our ability to perform causal or correlative analyses linking resistance to comprehensive patient characteristics. In addition, the single-center design of this study may limit the direct extrapolation of our findings to all regions of Yunnan. Future multi-center studies incorporating patients from border areas, rural clinics, and different healthcare tiers are warranted to confirm and expand upon these observations. The observed differences in resistance rates between groups should therefore be interpreted as associations, with the influence of potential confounding factors acknowledged. While we performed multivariable and stratified analyses, the sample size in certain subgroups was relatively small, which might affect the robustness of the findings. Thirdly, this study did not investigate the molecular mechanisms underlying the observed differences in drug resistance profiles between mono- and co-infected patients. Future research integrating virological studies, such as assessing the impact of HCV proteins on HIV replication fitness *in vitro*, is needed to elucidate the causal pathways. At last, as this was a cross-sectional study of patients with virological failure, we did not have access to pre-treatment plasma samples for baseline genotypic resistance testing. Therefore, we cannot distinguish between transmitted drug resistance present at therapy initiation and resistance acquired during treatment. Prospective studies with baseline and serial resistance testing are crucial to delineate the dynamics of resistance emergence in HIV/HCV co-infected patients. Despite these limitations, this study provides a comparison of the virological resistance landscape in two key patient populations at the point of treatment failure. The identified differences in resistance rates and mutation patterns offer a foundational virological perspective that warrants further investigation in prospective cohorts designed to integrate detailed clinical and behavioral data. Our findings, while primarily virological, suggest that HIV/HCV co-infection status may be associated with a distinct pathway to treatment failure. This underscores the importance of prompt genotype testing upon failure in all patients, and highlights co-infection as a potential clinical marker prompting vigilance.

## 5 Conclusion

This study characterizes the HIV-1 genotypic drug resistance landscape specifically among patients experiencing virological failure on first-line ART in Yunnan Province. Our findings indicate that HIV/HCV co-infection is associated with a significantly lower overall drug resistance rate compared to HIV-1 mono-infection in this treatment-failure population. Although multivariable analysis showed a marginal independent association, stratified analysis revealed that this difference is most pronounced during the early stages of virological failure (6–12 months) and among patients with moderate to severe immunodeficiency (CD4+ ≤ 350 cells/μL). Drug-resistant mutations, particularly those related to NNRTIs, remain the primary driver of HAART failure, with specific mutation patterns like T69D/N/S appearing less frequently in co-infected individuals. These results suggest that co-infection status may serve as a clinical marker for distinct resistance pathways. While these insights from a major referral center are relevant for high-prevalence settings in Southwest China, the conclusions reflect the need for broader, multi-center prospective studies to validate these associations and further elucidate the underlying virological mechanisms.

## Data Availability

The raw data supporting the conclusions of this article will be made available by the authors, without undue reservation.

## Footnotes

1
http://hivdb.stanford.edu
