# Supplementary material for: HIV-1 drug resistance among HIV/HCV co-infected patients with treatment failure in Yunnan, southwestern China: a cross-sectional study
Source: Front Microbiol. 2026 Mar 10;17:1715352. doi: 10.3389/fmicb.2026.1715352 (PMC13008895; doi:10.3389/fmicb.2026.1715352)
Supplement: Supplementary file 1 [file Table_1.docx]

Supplementary Table 1 Sequences of amplification primers and sequencing primers

| primer | sequence(5′→3′) | location (HXB2) direction |
| --- | --- | --- |
| MAW-26 | TTGGAAATGTGGAAAGGAAGGAC | 2028→2050 forward outer primer |
| RT21 | CTGTATTTCTGCTATTAAGTCTTTTGATGGG | 3539→3509 backward outer primer |
| PRO-1 | CAGAGCCAACAGCCCCACCA | 2147→2166 forward inner primer |
| RT20 | CTGCCAGTTCTAGCTCTGCTTC | 3462→3441 backward inner primer |
| DR1-S1^a^ | GCCAACAGCCCCACCA | 2151→2166  forward primer |
| DR1-S2^a^ | GGACCTACACCTGTCAAC | 2484→2501  forward primer |
| DR1-S3^a^ | CCTAGTATAAACAATGAGACAC | 2946→2967  forward primer |
| DR1-S4^a^ | GCTGGGTGTGGTATTCC | 3144→3128 reverse primer |
| DR1-S5^a^ | GTTCTAGCTCTGCTTC | 3456→3441 reverse primer |

a:sequencing primer, HXB2:the standard reference strain of HIV-1
